# Congenital microcephaly with early onset epileptic encephalopathy caused by *ASNS* gene mutation: A case report

**DOI:** 10.1097/MD.0000000000020507

**Published:** 2020-05-29

**Authors:** Chen Chen, Yunpeng Hao, Jianmin Liang, Xuncan Liu

**Affiliations:** a Department of Pediatric Neurology; b Department of Rehabilitation, The First Hospital of Jilin University, Changchun, Jilin, China.

**Keywords:** asparagine synthetase, asparagine synthetase, case report, congenital microcephaly, epileptic encephalopathy, gene mutation

## Abstract

**Rationale:**

Asparagine synthetase deficiency (ASNSD) refers to a congenital metabolic abnormality caused by mutation in the asparagine synthetase (*ASNS*) gene encoded by chromosome 7q21. Herein, we report the first case of ASNSD in China, in which novel *ASNS* mutations were identified.

**Patient concerns:**

A 6-month-old boy presented with a 4-month history of microcephaly and psychomotor developmental retardation and a 2-month history of epilepsy. Four months after birth, magnetic resonance imaging demonstrated a giant cyst in the right lateral ventricle, and a ventriculoperitoneal shunt was placed. Video electroencephalography showed a hypsarrhythmia pattern with a string of tonic-clonic and myoclonic seizures. On admission, physical examination showed microcephaly. Neurologic examination showed a decreased tension in the trunk muscles and an increased tension in the extremity muscles; tendon hyperreflexia was noted, and bilateral pathologic reflexes were positive.

**Diagnosis:**

A diagnosed of congenital microcephaly was made. Whole-exome sequencing revealed a heterozygous deletion mutation c.666_667delCT (p.L2221Lfs∗5) in exon 6 of the *ASNS* gene and a heterozygous missense mutation c.1424C>T (p.T457I) in exon 13 of the *ASNS* gene.

**Interventions:**

After admission, intravenous adrenocorticotropic hormone and oral topiramate was administrated for 4 weeks, while the seizures persisted. Then, levetiracetam and clonazepam were added.

**Outcomes:**

After the follow-up period of 18 months, video electroencephalography showed that complex episodes disappeared with changes in multiple focal spike and sharp waves; 1 focal attack arising from the left occipital region and 2 focal attacks arising from the right middle temporal and the right occipital region were recorded.

**Lessons:**

This is the first case of ASNSD in China. We identified 2 novel mutations (c.666_667delCT and c.1424C>T) in the *ASNS* gene, which expands the *ASNS* gene mutation profile and will be beneficial for genetic diagnosis.

## Abstract 2

Supplemental Digital Content is available in the text

## 1 Introduction

Asparagine synthetase deficiency (ASNSD) refers to a congenital metabolic abnormality caused by mutation in the asparagine synthetase (*ASNS*) gene encoded by chromosome 7q21.^[1]^ Clinically, ASNSD is characterized by dysplasia of the central nervous system and can present as congenital microcephaly, severe psychomotor developmental retardation, excessive irritability, refractory epilepsy, spasticity, dyskinetic quadriplegia, difficulty in feeding, and insufficient ventilation.^[2]^

Herein, we report the first case of ASNSD in China, in which novel *ASNS* mutations were identified.

## 2 Case presentation

**Figure 1:**
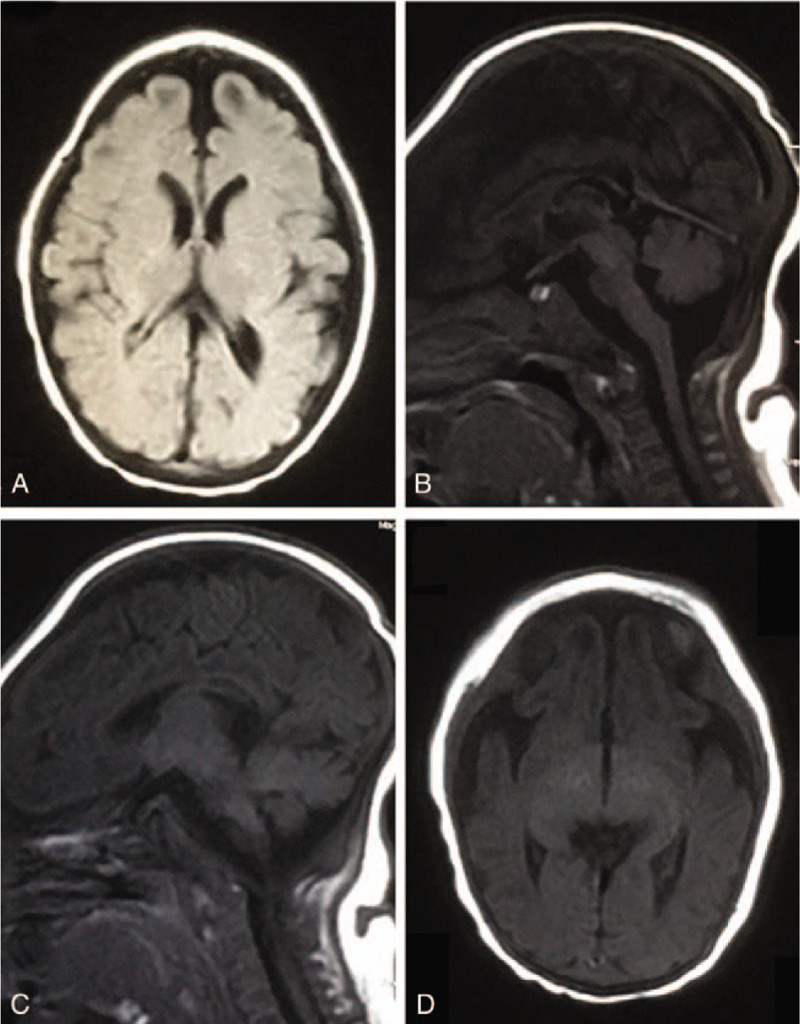
Brain magnetic resonance image (MRI) at 2 months after birth. Brain MRI showed dysplasia in the frontal lobes, corpus callosum, brainstem, and cerebellum along with widening of the cerebral sulci and gyrus.

**Figure 2:**
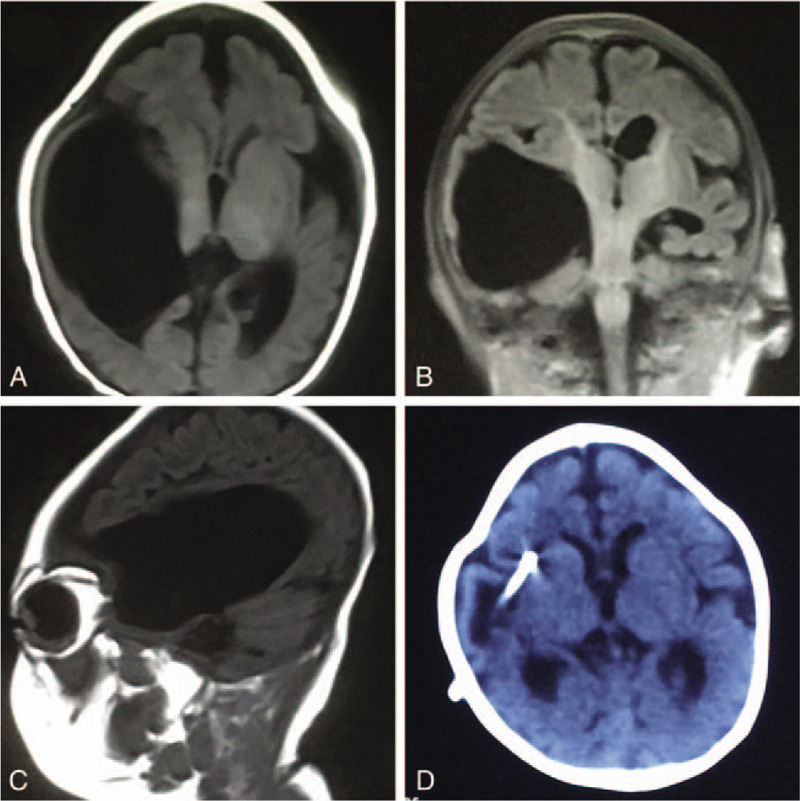
(A–C) Brain magnetic resonance image (MRI) at 4 months after birth. Brain MRI showed a giant cyst in the right lateral ventricle. (D) Head computed tomography (CT) at 6 months after birth: head CT showed the cyst was significantly smaller after 2 months of treatment.

A 6-month-old boy (Gravida 2, Para 2) presented to the Department of Pediatric Neurology with a 4-month history of microcephaly and psychomotor developmental retardation and a 2-month history of epilepsy. The boy was normally delivered at full term, and his mother experienced no abnormality during pregnancy. The postnatal Apgar score was normal; the birth weight was 3.6 kg (75% percentile of the standard weight), the birth height was 49 cm (25% of the standard height), and the head circumference was 32 cm (3% of the standard head circumference). Two months after birth, brain magnetic resonance imaging (MRI) showed dysplasia in the frontal lobes, corpus callosum, brainstem, and cerebellum, and the cerebral sulci and gyrus were widened (Fig. 1). Four months after birth, MRI demonstrated a giant cyst in the right lateral ventricle (Fig. 2 A–C), and a ventriculoperitoneal shunt was placed. Six months after birth, head computed tomography (CT) showed the cyst had shrank significantly (Fig. 2 D). Four months after birth, he developed seizures. The seizures manifested as repeated nodding with limb holding (5–7 times daily) or a single rapid shaking of the head and left lower extremity (≈20 times daily). Video electroencephalography revealed a series of tonic-clonic and myoclonic seizures; hypsarrhythmia was detected in the right occipitotemporal region. The parents also reported that the boy had a history of vomiting and choking. On admission, physical examination showed a weight of 5.2 kg (<1% of the standard weight), a height of 63 cm (1–3% of the standard height), and a head circumference of 35 cm (<1% of the standard head circumference) (Supplementary Fig. 1, http://links.lww.com/MD/E334). The bregma had closed. Neurologic examination showed a decreased tension in the trunk muscles and an increased tension in the extremity muscles; tendon hyperreflexia was noted, and bilateral pathologic reflexes were positive. Routine laboratory examinations were all normal. Whole-exome sequencing revealed a heterozygous deletion mutation c.666_667delCT (p.L2221Lfs∗5) in exon 6 of the *ASNS* gene and a heterozygous missense mutation c.1424C>T (p.T457I) in exon 13 of the *ASNS* gene. Further genetic investigation showed his father harbored the former deletion mutation and his mother had the latter missense mutation (Supplementary Fig. 2, http://links.lww.com/MD/E335). After admission, intravenous adrenocorticotropic hormone (ACTH; 10 units for the initial 2 weeks, and 20 units for the subsequent 2 weeks) and oral topiramate were administrated for 4 weeks, while the seizures persisted. Then, levetiracetam and clonazepam were added. After the follow-up period of 18 months, video electroencephalography showed that complex episodes disappeared with changes in multiple focal spike and sharp waves; 1 focal attack arising from the left occipital region and 2 focal attacks arising from the right middle temporal and the right occipital region were recorded.

## 3 Discussion

The ASNSD is a newly described rare autosomal recessive neurodevelopmental disease.^[1,3–5]^ In 2013, Ruzzo et al first reported 9 cases of ASNSD in 4 families with clinical manifestations of congenital microcephaly, developmental delay, progressive encephalatrophy, and refractory epilepsy; they confirmed ASNSD as a recessively inherited metabolic disorder caused by homozygous or complex heterozygous mutation(s) in the *ASNS* gene encoded by chromosome 7q21.^[1]^ Herein, we report the first case of ASNSD in China. Whole-exome sequencing revealed a heterozygous deletion/frameshift mutation c.666_667delCT (p.L2221Lfs∗5) in exon 6 of the *ASNS* gene and a heterozygous missense mutation c.1424C>T (p.T457I) in exon 13 of the *ASNS* gene. Both of these 2 mutations have yet to be identified (based on OMIM, HGMD and ClinVar databases).

The mutation of C.1424 C>A (p.T475N) in the *ASNS* gene has been recorded in the ClinVar database. However, this variant has not been published as a pathogenic variant, nor has it been reported as a benign polymorphism. The T475I variant of our patient had the same location of mutation, but different missense from above. So, the T475I variant is a strong candidate for a disease-causing variant; however, the possibility that it may be a rare benign variant cannot be excluded. Mutation c.666_667delCT results in a frame shift and induces a change in the amino acid sequence as well as an abnormal 3-dimensional structure of the protein. Although the c.666_667delCT (p.L2221Lfs∗5) mutation has not been previously reported, it may be near c.601delA (p.M201Wfs∗28), which has been reported and associated with microcephaly.^[6]^ It is not surprising that this protein is predicted to no longer be functional as an ASNS enzyme.

The *ASNS* encodes the protein asparagine synthetase, which is involved in the de novo synthesis of amino acid asparagine via transfer of ammonia from glutamine and aspartate.^[1,7]^ In patients with ASNSD, the reduced asparagine synthesis disturbs the proliferation and apoptosis of neuronal cells, thus leading to fetal cerebral dysplasia during pregnancy or after birth.^[8]^ The common clinical manifestations of ASNSD include microcephaly, severe psychomotor developmental delay, cortical blindness, hyperreflexia, encephalatrophy, increased limb muscle tone, decreased trunk muscle tone, feeding difficulties, and seizures. The seizure types include generalized tonic-clonic seizures, myoclonic seizures, tonic seizures, and complex partial seizures.^[2,3]^ Herein, we report the first case of ASNSD in China, and the clinical presentations were in accordance with previous reports.

Radiologically, ASNSD usually manifests as a small corpus callosum, a small pons, cerebellar dysplasia, ventricular dilatation, widened cerebral sulci and gyrus, and developmental retardation of myelin.^[2]^ However, intracerebral cysts are uncommon in ASNSD and were previously observed in only 1 case reported by Bensalem et al in 2015.^[9]^ In the present case, no cyst was observed on the MRI at 2 months after birth, while the repeated MRI at 4 months after birth showed a giant cyst in the right lateral ventricle. We speculate that ASNSD may be associated with progressive brain structural abnormalities, and dynamic radiologic observation should be highlighted.

There is no special treatment for ASNSD, and ASNS alternative treatment remains the first choice. Based on the cases reported to date, the majority of patients had developmental retardation (such as microcephaly) during the fetal period, and the efficacy of alternative treatment after clinical onset is limited. Thus, we recommend that alternative treatment should be initiated in the perinatal period. Furthermore, the mechanism by which ASNS passes the blood–brain barrier remains unclear; exogenous supplementation may not be able to increase the intracerebral ASNS concentration. Additionally, alternative treatment requires a high-dosage ASNS, which may disturb the adsorption of other amino acids and further aggravate the neurologic dysfunction. Alrifai and Alfadhel attempted ASNS alternative treatment in an Arab child with ASNSD, following which the patient's psychomotor development was significantly improved; however, the seizures were exacerbated and the treatment was terminated.^[10]^

## Author contributions

**Conceptualization:** Xuncan Liu, Yunpeng Hao, Jianmin Liang, Chen Chen.

**Formal analysis:** Chen Chen, Yunpeng Hao, Jianmin Liang.

**Investigation:** Xuncan Liu, Chen Chen.

**Writing – original draft:** Xuncan Liu, Chen Chen.

**Writing – review & editing:** Xuncan Liu, Yunpeng Hao, Jianmin Liang, Chen Chen.

## Supplementary Material

SUPPLEMENTARY MATERIAL
