# Supplementary figures and images for: Congenital microcephaly with early onset epileptic encephalopathy caused by ASNS gene mutation: A case report
Source: Medicine (Baltimore). 2020 May 29;99(22):e20507. doi: 10.1097/MD.0000000000020507 (PMC12245211; doi:10.1097/MD.0000000000020507)

**Supp. Figure 1.** Physical examination showed microcephaly.


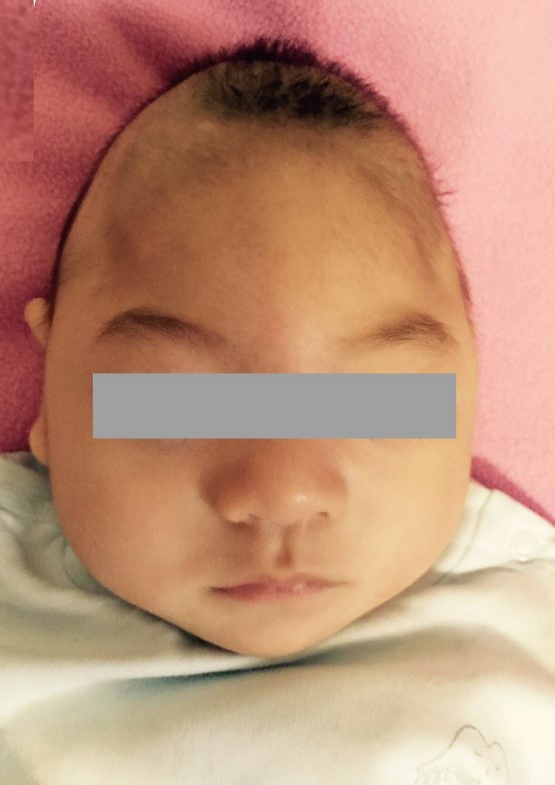

Supplement: SUPPLEMENTARY MATERIAL [file medi-99-e20507-s001.doc]
