# Supplementary material for: Congenital microcephaly with early onset epileptic encephalopathy caused by ASNS gene mutation: A case report
Source: Medicine (Baltimore). 2020 May 29;99(22):e20507. doi: 10.1097/MD.0000000000020507 (PMC12245211; doi:10.1097/MD.0000000000020507)

**Supp. Figure 2.** Genetic sequencing

Whole exome sequencing revealed a heterozygous deletion mutation c.666_667delCT (p.L2221Lfs*5) in exon 6 of the *ASNS* gene, which was shared with his father (A), and a heterozygous missense mutation c.1424C>T (p.T457I) in exon 13 of the *ASNS* gene, which was shared with his mother (B).


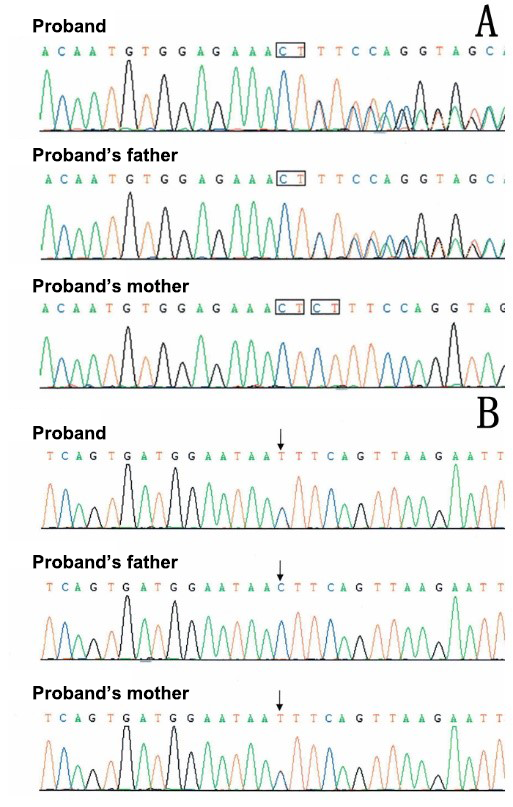

Supplement: SUPPLEMENTARY MATERIAL [file medi-99-e20507-s002.doc]
